# Marital dissolution and associated factors in Hosanna, Southwest Ethiopia: a community-based cross-sectional study

**DOI:** 10.1186/s40359-023-01051-3

**Published:** 2023-01-25

**Authors:** Likawunt Samuel Asfaw, Getu Degu Alene

**Affiliations:** College of Medicine and Health Sciences, School of Public Health, Bahir Dar University, Bahir Dar, Ethiopia

**Keywords:** Divorce, Family, Marital dissolution, Marriage, Separation

## Abstract

**Background:**

Marriage dissolution, divorce, or separation from a spouse or common-law partner is a serious public health concern due to its increasing prevalence and devastating health and socio-economic consequences. Evidence suggests an increased risk of marital instability in Ethiopia. In addition, the extent of marital dissolution and other related factors have increased in the study area. Despite these, the prevalence of marital dissolution and the influence of associated factors (main reason for marriage, and parental history of marital dissolution) on marital dissolution has not been assessed in the study area. Therefore, the aim of this study was to investigate the prevalence of marital dissolution and its associated factors among residents of Hosanna town in southwestern Ethiopia in 2022.

**Methods:**

We conducted a community-based cross-sectional study among 459 randomly selected Hosanna Township residents. We used structured questionnaires to collect data. Descriptive statistics and logistic regression were performed to describe the data and test-associated factors, respectively. A p-value less than 0.05 was used to define statistical significance. We used STATA 14 and IBM SPSS 25.0 computer packages to process data.

**Results:**

Out of the 459 potentially eligible individuals, 450 participants properly responded to the questionnaires yielding a response rate of 98.04%. Of these, 218 (52.9%) were female. The commonly reported reason for marriage was to have children 150 (36.9%). The prevalence rate of marital dissolution was 26.0% (95% CI: (21.7%, 30.3%)). The participant's level of education and the primary reasons (motives) why they get married were statistically significantly associated with marital dissolution. The odds of marital dissolution was higher among participants who completed secondary education (AOR = 3.2, 95% CI = 1.26–8.17) compared to those having no formal education. The participants who married for companionship reasons (AOR = 0.31, 95% CI = 0.11–0.83) had significantly lower odds of marriage dissolution compared with those who married for financial security.

**Conclusions:**

In this study, the prevalence of marital dissolution was high. The participant’s level of education and the primary reasons (motives) why they getting married were significantly associated with marital dissolution. Therefore, an integrated, community-based approach should be developed to prevent marital dissolution.

## Background

Marital dissolution, a break in the continuity of the matrimonial bond is appealed to be a serious public health challenge in the world [1–3]. It is a serious issue due to its rising prevalence and devastating health and socio-economic consequences. In earlier times, marriage was recognized as a lifetime commitment and ended due to the death of either of the partners [4]. However, empirical research suggests that the world is in a state of transition in the characteristics of bond formation and marriage dissolution [5]. Moreover, there is a growing acceptance of marital dissolution, weakening of marital bonds, and increased opportunity for marital instability [5]. Consequently, the magnitude of marital dissolution is rising Worldwide [6, 7].

Marital dissolution is one of the major social determinants of health and attributes to the majority of chronic physical and mental health disorders [1, 8]. It has been linked to worsening mental and physical health problems [1, 8]. Marriage dissolution affects health in different ways. Like most stressors, marital discord can lead to the production of stress hormones, which can lead to chronic systemic diseases [9]. More commonly, anxiety, depression, and cardiovascular disorders are the commonly reported physical and mental health problems after marriage dissolution [1, 8, 10]. In addition to physical and mental illnesses, it is also known to be associated with injuring oneself with the intent to die. A previous study [11] speculated that marital dissolution is associated with an increased risk of suicidal ideation. It has copious consequences on children's psychological and physical well-being [12].

Furthermore, it exposes people to health risk behaviors such as changes in eating patterns and heavy alcohol consumption [13], and multiple sexual partners [14, 15]. Correspondingly, having multiple sexual partners seems to be a common problem that divorced/separated women were more likely to report five or more lifetime sex partners than never-married women [16].

Determining the rate of marital dissolution and potential associated factors is important for developing strategies to reduce the risk of divorce. Furthermore, it is used to notify researchers, programmers, and policy-makers about the burden of marital dissolution, thereby supporting the process of mitigating the problem [17]. Previous researchers have found inconsistent findings on the proportion of marital dissolution and the effect of partners' socio-demographic characteristics on the risk of marital dissolution [1, 16, 18, 19]. Moreover, most previous studies have used secondary data and have focused on divorce, not separation [18, 20, 21].

With the above issues in mind, this study assessed the factors associated with marital dissolution. The variables considered as associated factors in this study were the parental marital dissolution history, marital-related characteristics of spouses (the main reason for getting married, how many times have marriage concluded), and the information (knowledge and skill) about their marital relationship. These variables were selected based on their association with marital dissolution [22–25].

Based on prior studies [24, 26], we hypothesized that parents' divorce/separation history has an association with their children's marital dissolution suggesting that couples whose parents were divorced/separated had a higher risk of marital dissolution compared to couples whose parents were not divorced or separated. Empirical studies [24, 27] have shown that most children of divorced/separated parents experience emotional and communication problems, lack of commitment and self-confidence in marriage [24, 27]. These can subsequently harm future child marriage relationships. However, previous studies did not show the association between parental divorce and children's marital dissolution but rather the effects of parental divorce on children's behavior.

Furthermore, we hypothesize that the main reason for marriage is related to the dissolution of the marriage. A previous study identified that, one of the reasons related to marital dissolution was premarital reasons such as the clear purpose of marriage and the reasons for it [28]. The specific reasons (for financial reasons, to get children, for companionship, and or for falling in love) and their relationship to the marital dissolution have not been assessed.

In addition, we also hypothesize whether the number of marriages (first, second, third, etc.) contributes to the dissolution of marriage. Previous studies have assessed the association between the number of marriages and reported inconsistent results. Compared with first-married adults, remarried adults have positive attitudes toward divorce and are more likely to file for dissolution when experiencing marital distress [24]. However, another empirical study reported that the association between the number of marriages and marriage dissolution was questionable and suggested future research [25].

This study also hypothesizes that the marital information (knowledge and skills) possessed by partners is related to marital dissolution. Previous research has shown that factors such as lack of knowledge about marriage and relationship-building skills are significant predictors of marital breakdown [29]. It was also noted that married people who did not have basic information about their relationship experienced more problems in their marriage [30].

The effects of some of the aforementioned factors such as parental divorce/separation history, the main reason for marriage, and the number of marriages (first, second) were not assessed at the population level in the study area. Therefore, the aim of this study was to investigate the prevalence of marital dissolution and its associated factors among residents of Hosanna town in southwestern Ethiopia.

## Methods

### Study design

A community-based cross-sectional study was conducted to assess the prevalence (point-prevalence) of marital dissolution among Hosanna residents.

### Study setting

This study was conducted in Hosanna town. Hosanna is the administrative and commercial center of the Hadiya Administrative Zone in the Southern regional state of Ethiopia. It is located 232 km South West of Addis Ababa. As for demographic characteristics, the population of Hosanna has been rapidly growing since its inception. According to the housing and population census of Ethiopia, the population of the Town was 13,467 in 1984, and (31,701) ten years later in 19,944 [31]. In 2007, Hosanna had a population of 69,995 people [31]. According to Hosanna Municipality and the Ethiopian Demographic and Health Survey [31], the town of Hosanna is divided into six *Kebele* (smaller administrative units in Ethiopia). We used this pre-arranged structure of the town for the current study.

In Ethiopia, only Twenty-seven percent of women aged 15–49 have never married and 11 percent are divorced. Correspondingly, less than one percent of women aged 45–49 have never been married indicating that both marriage and marital dissolution are universal in Ethiopia [32]. Recruitment of participants and data collection were carried out from February 1 to March 30, 2022.

### Participants

Individuals who lived in Hosanna town and were able to answer specific marital questions were included in this study. We used marital-specific questions such as the “current marital status,” “the prime reasons for getting married”, “age at marriage”, and “parental marital dissolution history”, to consider people as eligible for this study. These variables were considered because they were associated with marital dissolution, which was the outcome variable of this study. The prospective study participants were recruited from the framed source population. Those who met the eligibility criteria were included in the study population. We obtained the data of details of the existing government structure, the number of Households, and the total number of people living in each administrative unit of the Town from Hosanna Town Municipality, *Kebele* Administrations, and Urban Health Extension workers. The research team included field supervisors and data collectors.

The first eligibility criterion was living in Hosanna Town. Since this study was carried out in Hosanna, people who have been living in Hosanna Town for at least six months were included in the current study. We assessed this criterion by asking participants. The ever-married individuals with a marriage history: currently married (in marital union during the data collection period), divorced, or widowed were included in the study without age and sex restriction. In contrast, a few individuals who refused to participate in the study and were unable to provide adequate information due to health problems at the time of data collection were excluded from the study.

### Variables and definitions

The prevalence of marital dissolution is the outcome of this study. The prevalence of marital dissolution was determined from the study sample. In other words, we estimated the point prevalence of marital breakdown (divorce or sedation) from the study sample that was identified during the data collection period. The marital specific characteristics prime reasons to get married, age at marriage, type of marriage, forms of marriage, parental marital dissolution history, General health status (presence of known chronic illness), behavioral correlates(substance use, Communication problem, conflicting behavior, marital commitment), and socio-demographic factors(sex, religion, level of education, house ownership) were the independent variables.

In this study, marriage was defined as the legal or formal union of two people (of the opposite sex), a man and a woman, as partners in a personal relationship. Moreover, divorce was defined as the legal termination of a marriage, the separation of husband and wife which confers on the parties the right to remarriage according to the laws of each country [33, 34]. Whereas, separation refers to the termination of a marriage on the basis of civil, religious, and/or other traditional provisions without conferring on the parties the right to remarry [33, 34]. Correspondingly, marital dissolution is defined as the termination of a marital relationship as a result of divorce or separation [33, 34].

### Data sources and measurement

In this study, we collected raw data directly from the study participants. Thus, the study participants were the data sources. Data were collected using a series of forms completed using face-to-face interview techniques. The form includes demographics, general health, and marriage-specific characteristics.

Data were collected using a structured questionnaire. A questionnaire was developed by reviewing relevant literature [22, 23, 33–39]. It was prepared in English and translated into Amharic. The questionnaire had three parts, the first part contained four questions and was about the socio-demographic characteristics of the individual participants, the second part contained three questions designed to collect data on general health and health risk behavior, and the third part contained eleven items designed to assess the marital specific characteristics and marital dissolution.

The questionnaire was pre-tested on 10% of the sample in an adjacent Town (Durame, capital town of Kembata Zone) where the study didn’t take place. Therefore, a modification was made based on the results of the pre-test, and the modified version was used for actual data collection. The internal consistency of the items in the questionnaire was acceptable with the value of Cronbach’s alpha (0.71), exceeding the index of 0.7 [40, 41].

Prior to data collection, the data collectors and supervisory teams contacted officials of each administrative unit with official letters. The households and individual participants were selected with the help of a guide from the respective administrative units. After contacting these individuals, the details of the concern of the team were explained to each participant by the assigned team leader, and the process of informed consent was secured.

Prior to the participant recruitment and data collection process, the research team received two days of training (basic principles of research ethics, data collection tools, and the roles and responsibilities of each team member). Six first-year health science students collected data. Two public health professionals were assigned to supervise the data collection process. The investigators of the research project coordinated the entire fieldwork.

### Bias

Depending on the study design different techniques were undertaken to ascertain both the selection and information bias in this study. In the selection stage, the study participants were randomly selected based on the pre-determined criteria and included in the study. During the data collection process, revisits were scheduled to complete the missed data and reduced the information bias [42]. Furthermore, several individuals participated in the data collection to enhance the depth of the findings. The training was given to data collectors to familiarize them with the local culture, research instruments, and principles of research ethics. Statistical procedures were also performed to treat information bias due to missing data [42].

### Study size and sampling techniques

We used a single population proportion formula to determine the number of individuals to be included in the study [43] that is appropriate for the estimation of a single proportion [44]. The proportion of marital dissolution was obtained from a previous study (45%) [20], estimated with 95% confidence and 5% precision, and took into account a 20% non-response rate to determine the sample size. Consequently, the sample size was 459.

**Fig. 1:**
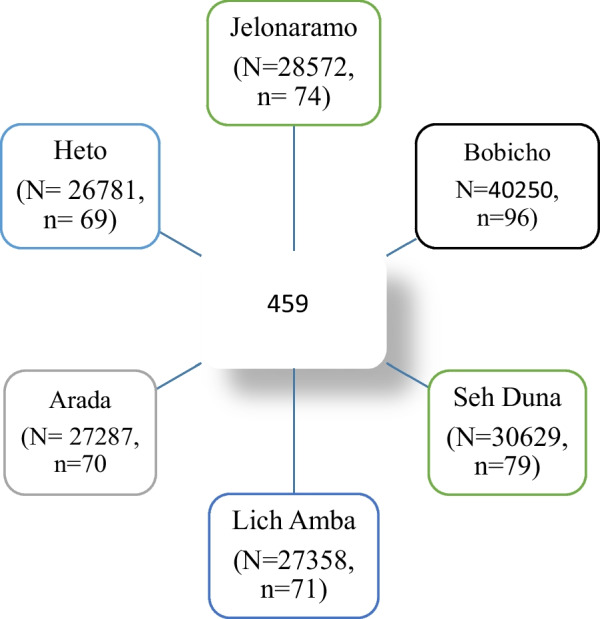
Schematic presentation for the sampling procedure of the study “Marital dissolution in Hosanna town, Southwest Ethiopia: A community-based cross-sectional study”, 2022

This sample size was proportionally allocated to each *Kebele* based on the number of households in each *Kebele* (Fig. 1). The town of Hosanna is divided into six *kebels*. We used this pre-arranged structure of the town to frame the current study. We used the STAT CALC program of the EPI INFO statistical package to calculate the sample size.

A simple random sampling technique was employed to select the study participants. The list of Households in each sub-cities that were documented in the respective administrative units was used to select the study participants. We first randomly assign a numeric code from one to six to each of the six sub-cities. Arada 1 is the first English alphabet letter, Bobicho 2, Heto 3, Sech Duna 6, and so on. Depending on the number of Households in each Sub-cities, we assigned four-digit alpha-numeric codes for each household. For example in Sech Duna Kebele there are three thousand six hundred and twenty-nine registered Households, so, the code for the first Household was 6 and the code for the last Household was 30,629. Correspondingly, there were forty-thousand two hundred and fifty Households in Bobicho *Kebele*, the code for the first Household was 2 and the code for the last Household was 40,250. A similar procedure was followed to code and select study participants in all *Kebele*.

Finally, among all households, randomly selected households using computer-generated numbers were included in the study. In situations where there was more than one person who satisfy the inclusion criteria in a given household, an individual was selected using a simple random technique (lottery method) and included in this study These codes, which we posted on the existing family archives, were removed as soon as the data collection process was completed.

### Statistical methods

The collected data were coded, cleaned, and entered into IBM SPSS 25 (International Business Machines Corporation (IBM) Statistical Package for the Social Sciences (SPSS) for Windows version 25 for analysis. We described the sample using frequencies, percentages, and diagrams. The distribution of the data set was tested using statistical tests (Shapiro–Wilk test, and Kolmogorov–Smirnov test) and graphical (histogram) methods [45].

We used logistic regression (bivariate and multivariate) analysis to assess whether there was a significant association between the associated factors and dependent variables. Before fitting the final model and reporting the results, we performed the necessary evaluations, including Multicollinearity and goodness-of-fit tests. Therefore, the Variance Inflation Factors (VIFs) test was used to assess the Multicollinearity among the independent variables, and those that showed no Multicollinearity were fitted to the multivariable logistic regression model through a backward stepwise method to reduce the effects of cofounders. The variables with a p-value of < 0.2 in the Bivariable analysis were considered for multivariable logistic regression analysis.

The Hosmer–Lemeshow goodness-of-fit statistic was used to check the model fit. We used the adjusted odds ratio with a 95% confidence interval to examine the strength and direction of the association between the independent variable and the outcome variable. A P-value of less than 0.05 [46] was used to define statistical significance. Finally, the findings were presented in the form of tables, graphs, and text. STATA 14 software package (Stata Corporation, College Station, Texas, 77,845, USA) and IBM SPSS 25.0 was used for data analysis.

## Results

### Participants

Out of the 459 potentially eligible individuals, 450 participants responded to each item listed in the questionnaires yielding a response rate of 98.04%.Overall, the data of 450 participants were included in the analysis. Of these, 218 (52.9%) were women. The age of the study participants was measured in years with a mean age of 36.91 years and a standard deviation (SD) of 6.67 years. Details of the background characteristics of participants are depicted in Table 1 below,

**Table 1:** Summary of the descriptive statistics of the study participants expressed as the mean (± standard deviation) or number (%)

Study variables	Male (n = 212)	Female (n = 238)	Total (n = 450)
Numerical variable			
Age (years)	36.51 ± 6.82	37.27 ± 6.54	36.91 ± 6.67
Categorical variables			
Religion			
Orthodox	51 (24.1)	72 (30.3)	123 (27.3)
Protestant	121 (57.1)	116 (46.7)	237 (52.7)
Islam	29 (13.7)	29 (12.2)	58 (12.9)
Catholic	8 (3.8)	13 (5.5)	21 (4.7)
Others	3 (1.4)	8 (3.4)	11 (2.4)
Level of education			
No formal education	19 (9.0)	17 (7.1)	36 (8.0)
Primary school	57 (26.9)	54 (22.7)	111 (24.7)
Secondary school	73 (34.4)	68 (28.6)	141 (31.3)
Higher education	63 (29.7)	99 (41.6)	162 (36.0)
Perceived health status			
Very poor	13 (6.1	11 (4.6)	24 (5.3)
Poor	30 (14.2	44 (18.5)	74 (16.4)
Good	82 (38.7)	90 (37.8	172 (38.2)
Very good	60 (28.3)	63 (26.5)	123 (27.3)
Excellent	27 (12.7)	30 (12.6)	57 (12.7)
Having chronic illness			
No	177 (83.5)	193 (81.1)	370 (82.2)
Yes	35 (16.5)	45 (18.9)	80 (17.8)
Current substance use			
No	176 (83.0)	197 (82.8)	373 (82.9)
Yes	36 (17.0)	41 (17.2)	77 (17.1)

### Marital-related characteristics of study participants

Of a total of 450 participants, 407 (90.4%) had a marriage history, from which the majority of them 241(59.2%) did not get marital information regarding the reasons for getting married; 150(36.9%) of study participants chose to get married to have children. Two hundred and fourteen (52.6%) of ever-married participants reported marital conflicts and conflicts often occur during illness72 (33.6%) and holidays.72 (33.6%) (Table 2).

**Table 2:** Summary of marital-related characteristics of ever-married (n = 407) study participants

Study variables	Male (n = 189)	Female (n = 218)	Total (n = 407)
Ever get marital information (n = 407)			
Yes	80 (42.3)	86 (39.4)	166 (40.8)
No	109 (57.7)	132 (60.1)	241 (59.2)
Source of marriage information (n = 166)			
Mass media	7 (7.8)	11 (11.3)	18 (9.6)
Religious educators	29 (32.2)	30 (30.9)	59 (31.6)
Family	27 (30.0)	19 (19.6)	46 (24.6)
Friends	13 (14.4)	21 (21.6)	34 (18.2)
Others	14 (15.6)	16 (16.5)	30 (16.0)
Inspiration to get married (n = 407)			
Self	109 (57.7)	118 (54.1)	2227 (55.8)
Family	57 (30.2)	61 (28.0)	118 (29.0)
Friend	16 (8.5)	37 (17.0)	53 (13.0)
Others	7 (3.6)	2 (0.9)	9 (2.2)
The main reason to get married (n = 407)			
For financial security	62 (15.2)	31 (14.2)	93 (22.9)
To get kids	68 (36.0)	82 (37.6)	150 (36.9)
For companionship	29 (15.3)	59 (27.1)	88 (21.6)
For social security	29 (15.3)	44 (20.2)	73 (17.9)
Other reasons¶	1 (0.5)	2 (0.9)	3 (0.7)
Number of marriage(n = 407)			
Once	174 (92.1)	191 (87.6)	365 (89.7)
Twice	14(7.4)	26 (11.9)	40 (9.8)
More than two times	1(0.5)	1(0.5)	2 (0.5)
Ever had a conflict (n = 407)			
No	94 (49.7)	99 (45.4)	193 (47.4)
Yes	95 (50.3)	119 (54.9)	214 (52.6)
Circumstances conflicts commonly occur in couples (n = 214)			
Illnesses in the family	29 (30.5)	43 (36.1)	72 (33.6)
During holidays	36 (37.9)	36 (30.3)	72 (33.6)
During Pregnancy	6 (6.3)	16 (13.4)	22 (10.3)
Making major decisions	7 (7.4)	10 (8.4)	17 (7.9)
Others	17 (17.9)	14 (11.8)	31 (14.5)

¶ = for religious beliefs, to secure a public commitment.

### The prevalence of marital dissolution

**Fig. 2:**
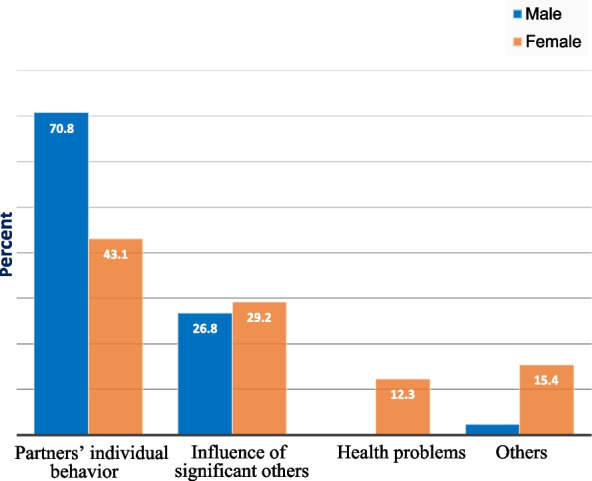
Reasons for marital dissolution expressed in percent (%)

The analyses of the prevalence of marital dissolution in the total sample suggest that 106 (26.0%) [95% CI: (21.7, 30.3)] of study participants reported that they had experienced marital dissolution. The proportion was 41 (38.7%) for males and 65(61.3%) for female participants. Correspondingly, the prevalence of marital dissolution is higher in those who reported no marital education (65 (61.3%)) compared to participants who ever had marital education (41 (38.7%)). The 57 (53.8%). participants reported the individual partners' behavioral factors as a major reason for marital dissolution (Fig. 2). Of those who experienced marital dissolution, 68.9% of them reported no parental history of marital dissolution and 28.3% had a parental history of marital dissolution.

### Logistic regression findings

The p-value of the final model is greater than the conventional threshold (0.05), indicating that the model fits well (chi-square = 4.323, P-value = 0. 827) [39],^47^. A VIF closer to 1 for a given range of independent variables (1.01–1.05) indicates the absence of Multicollinearity among the predictors in the model [48].

The binary logistic regression analysis showed that the participant’s level of education and the primary reasons (motives) why they got married were statistically significantly associated with marital dissolution In multivariate analysis (Table 3), participants who had completed secondary education had higher odds of marital breakdown than those with no formal education (AOR: 3.2, 95% CI 1.26–8.17). On the other hand, although more women reported marital dissolution, the association was not statistically significant (P-value = 0.24). Nonetheless, participants who married for companionship reasons (AOR = 0.31, 95% CI = 0.11–0.83) had significantly lower odds of marital dissolution compared with those who married for financial security.

**Table 3:** Binary logistic regression findings of factors associated with marital dissolution, 2022

Characteristics	Marital dissolution		AOR	[95% CI]	P value
	Yes, N (%)	No, N (%)			
Sex					
Male	41 (38.7)	148 (49.2)	1.35	0.81, 2.23	0.24
Female	65 (61.3)	153 (50.8)	1		
Highest level of education					
No formal education	12 (2.9)	19 (6.3)	1		
Primary education	25 (23.6)	75 (24.9)	1.85	0.73, 4.69	0.19
Secondary School	22 (20.8)	104 (34.6)	3.2	1.26, 8.17	*0.01*
Higher education	47 (44.3)	103 (34.2)	1.3	0.58, 3.33	0.45
The main reason to get married					
For financial security	6 (5.7)	87 (28.9)	1		
To get child	69 (65.1)	81 (26.9)	0.08	0.03,0.25	< *0.001*
For companionship	19 (17.9)	69 (22.9)	0.31	0.11,0.83	0.02
For social security	11 (10.4)	62 (20.6)	0.43	0.14,1.25	0.12
Others	1 (0.9)	2 (0.7)	0.11	0.01.1.53	0.1

N = Number of observations, 1 = Reference, CI = Confidence Interval, AOR = Adjusted Odds ratio.

## Discussion

This study aimed to investigate the prevalence of marriage dissolution among married people in Hosanna Township. The prevalence of marital dissolution in this study area was 26.0%, which was comparable to that of study in Ethiopia, where approximately 25% of married women were reported to be divorced from their first relationship [18].

However, the results of this study was lower than the reported rate of marital dissolution in Ethiopia. 45% of first marriages in Ethiopia end in divorce [20]. Furthermore, this finding is much higher than the Ethiopian Demographic and Health Survey report (2019), which showed that 6% of women (15–49 years) in Ethiopia were divorced or separated in Ethiopia [32].

Differences in the methods used, as well as the composition of the study populations, could explain the observed discrepancies between previous and current findings. For example, previous studies used National Family and Fertility Survey data and restricted [15–49] group [20]. This finding builds on existing evidence showing the prevalence of marriage dissolution remained high in Ethiopia.

This finding raises concerns over the social tie in the community. Marriage is a huge social institution [49] and a foundation for society [22]. It is concluded through religious doctrines, traditional ceremonies (customs of the society in which it is found) [50], or a public act [51]. It is governed by the codes of ethics and family law [35]. On the other hand, divorce, or marital dissolution is a judicially administered process that legally terminates a marriage that permits remarrying [35]. So, a disturbance in this institution indirectly reflects the weakening of the social tie and the erosion of the entire system surrounding marital relations in Ethiopia.

Further, the cross-tabulation analysis revealed that more women (61.3%) than men (38.7%) reported marital dissolution. The logistic regression findings revealed a negative relationship between the highest level of education and marital dissolution, meaning that people who have completed secondary education were more likely to have marital dissolution compared to those who had no formal education. This finding is consistent with previous reports showing how education level affects the presence of marriage dissolution [36, 37]. Again, this study was consistent with previous reports in Ethiopia [39].

In contrast to this, a previous study reported that individuals who did not attend formal education had higher odds of experiencing marital dissolution [18]. Recent research has also concluded that marriages with at least one highly educated partner are less likely to divorce than uneducated couples [52]. Furthermore, another study in rural South Africa indirectly revealed the effect of education on marriage dissolution and reported that those with higher education were more likely to remain in one marriage than those who had never attended school [19]. Being limited to women and methodological differences between the previous and the current study could more likely explain the observed differences [18].

The negative aspects of education, for instance, giving more credit to scientific issues over social and religious values, the tendency to look at more options, the need not want to live deprived of one's rights, and coping with post-divorce/separation social and economic problems, etc., could more likely explain the negative effects of education on marital dissolution. Most distressed couples prefer to stay together rather than file for marital dissolution due to fear of the negative consequences of marital dissolution on their life and children [53].

In Ethiopia, couples who wish to divorce or separate are notified "You considered the consequences before you rush to divorce/separate?" by their family, court judges, and friends. Educated people will be better able to cope with the social and economic consequences of marital dissolution. Second; it is well-known that people with higher education tend to understand their roles and responsibilities [54]. Whereas, if either or both spouses fail to fulfill their obligations and put undue pressure on each other, the marriage may end in divorce or separation.

In addition to the effect of level of education, the present study also assessed the association between the circumstances of entry into marriage (prime reason to get married) and marital dissolution and identified that the proportion of marital dissolution was significantly lower among participants who get married to get children and for companionship reasons compared to those who get married with motives (such as for financial security (arranged marriage)).

We were unable to find similar studies that directly described the effects of arranged marriages (marriages entered into for financial security) on marriage dissolution. However, previous studies consistently reported the negative effect of “arranged marriages” (marriages proposed by other people for various reasons than by mates for love and companionship [23] on marital dissolution The risk of divorce was more common among residents whose marriages were arranged.

On the other hand, romantic marriages were stable because they were based on affection between the two individuals [38]. It is generally observed that the risk of marital dissolution was low among partners who get marry for love and companionship compared to those for financial security. Marriage is one of the ancient and socially acceptable companionships between a man and a woman, which is regulated by beliefs, customs, laws, and attitudes that prescribe the rights and duties of the partners [33, 55]. It must then be based on love and the free will of the couple. In contrast, pre-arranged and money-oriented marriages should be discouraged. The strength of this study is that it was based on a simple random sampling technique and therefore, the findings can be generalized to the studied population [56]. The limitation of the present study is that the temporal link between the outcome and the exposure cannot be established because data were collected at one point in time.

## Conclusions

In conclusion, Ethiopia has a high marital dissolution rate. A partner’s level of education and the primary reasons surrounding entry into marriage are the predictors of marital dissolution. Preventing arranged marriages, such as those for economic security, and developing strategies to balance marital relationships and education will reduce the rate of marriage dissolution in Ethiopia.

Notably, this research highlights the fact that unless corrective measures are taken, marital dissolution is likely to escalate further. This event infers the need for urgent and integrated actions to assure strong, happy, and enduring marriages. Society is also recommended to discourage marital dissolution and arranged marriages (marriages for social, economic, or other purposes than for love and companionship between the partners) to increase the likelihood that people have an enduring and presumably happier family life.

## Data Availability

The datasets (SPSS, STATA, and data collection tools) used and/or analyzed during the current study will be available from the corresponding author upon reasonable request.

## Abbreviations

AOR: Adjusted odds ratio
CI: Confidence interval
IBM: International business machines corporation
SD: Standard deviation
SPSS: Statistical package for social sciences
STATA: Stata Corporation, College Station, Texas, 77,845, USA
VIFs: Variance inflation factors

## References

[1] Ding D, Gale J, Bauman A, Phongsavan P, Nguyen B (2021). Effects of divorce and widowhood on subsequent health behaviours and outcomes in a sample of middle-aged and older Australian adults. Sci Rep..

[2] Meyer JM, Percheski C (2017). Health behaviors and union dissolution among parents of young children: Differences by marital status. PLoS ONE.

[3] Jk K-G (2018). Marriage, divorce, and the immune system. Am Psychol..

[4] Stanley SM, Rhoades GK, Whitton SW (2010). Commitment: functions, formation, and the securing of romantic attachment. J Fam Theory Rev.

[5] Wagner M. On increasing divorce risks. 2020. 37–61 p.

[6] Guarneri A, Rinesi F, Fraboni R, De Rose A (2021). On the magnitude, frequency, and nature of marriage dissolution in Italy: insights from vital statistics and life-table analysis. Genus..

[7] Kennedy S, Ruggles S (2014). Breaking up is hard to count: the rise of divorce in the United States, 1980–2010. Demography.

[8] Wójcik G, Zawisza K, Jabłońska K, Grodzicki T, Tobiasz-Adamczyk B. Transition out of marriage and its effects on health and health–related quality of life among females and males. COURAGE and COURAGE-POLFUS–Population Based Follow-Up Study in Poland. Vol. 16, Applied Research in Quality of Life. 2021. 13–49 p.

[9] Rodriguez AJ, Margolin G (2013). Wives’ and husbands’ cortisol reactivity to proximal and distal dimensions of couple conflict. Fami Process.

[10] Matthew E, Dupre AN (2016). Marital history and survival after a heart attack. Soc Sci Med.

[11] Kõlves K, Ide N, De Leo D (2010). Suicidal ideation and behaviour in the aftermath of marital separation: Gender differences. J Affect Disord.

[12] Garriga A, Pennoni F (2020). The causal effects of parental divorce and parental temporary separation on children’s cognitive abilities and psychological well-being according to parental relationship quality. Soc Indic Res..

[13] Reczek C, Pudrovska T, Carr D, Thomeer MB, Umberson D (2016). Marital histories and heavy alcohol use among older adults. J Health Soc Behav.

[14] Peterson GW, Bush KR. Handbook of marriage and the family: Third edition. Handbook of Marriage and the Family: Third Edition. 2013. 1–914 p.

[15] Eng PM, Kawachi I, Filzmaurice G, Rimm EB (2005). Effects of marital transitions on changes in dietary and other health behaviours in US male health professionals. J Epidemiol Community Health.

[16] Liddon N, Leichliter JS, Habel MA, Aral SO (2010). Divorce and sexual risk among US women: findings from the national survey of family growth. J Women’s Heal..

[17] Harder T. Some notes on critical appraisal of prevalence studies: comment on: “the development of a critical appraisal tool for use in systematic reviews addressing questions of prevalence. Int J Heal Policy Manag. 2014; 3(5): 289–90. 10.15171/ijhpm.2014.99 10.15171/ijhpm.2014.99 PMC4204748 25337603

[18] Dagnew GW, Asresie MB, Fekadu GA, Gelaw YM (2020). Factors associated with divorce from first union among women in Ethiopia: Further analysis of the 2016 Ethiopia demographic and health survey data. PLoS One..

[19] Batidzirai JM, Manda SOM, Mwambi HG, Tanser F (2020). Discrete survival time constructions for studying marital formation and dissolution in rural South Africa. Front Psychol.

[20] Tilson D, Larsen U (2000). Divorce in Ethiopia: The impact of early marriage and childlessness. J Biosoc Sci.

[21] Mekonnen KY, Ayalew K (2019). Prevalence, causes and consequences of divorce in bahir Dar city, Ethiopia. Ajsw..

[22] Bethmann D, Kvasnicka M (2011). The institution of marriage. J Popul Eco.

[23] Gupta GR (1976). Love, arranged marriage, and the Indian social structure. J Comp Fam Stud..

[24] Whitton SW, Stanley SM, Markman HJ, Johnson CA (2013). Attitudes toward divorce, commitment, and divorce proneness in first marriages and remarriages. J Marriage Fam.

[25] Zahl-Olsen R (2023). Understanding divorce trends and risks: the case of Norway 1886–2018. J Fam Hist.

[26] Costello D. Effects of divorce on future relationships. ESSAI. 2003;1(Article 13.):31–3. https://www.verywellfamily.com/effects-of-divorce-on-teens-2609530

[27] Whitton SW, Rhoades GK, Stanley SM, Markman HJ (2008). Effects of parental divorce on marital commitment and confidence. J Fam Psychol..

[28] Manning WD, Cohen J (2012). Premarital cohabitation and marital dissolution: an examination of recent marriages. J Marriage Fam.

[29] Rijavec Klobučar N, Simonič B (2018). Risk factors for divorce in Slovenia: a qualitative study of divorced persons’ experience. J Fam Stud.

[30] Sanizah A., Hasfariza F., S. Norin Rahayu ANNN. Determinants of marital dissolution: a survival analysis approach. Int J Econ Stat. 2014;2(January 2014).

[31] Central Statistical Agency (CSA) Ethiopia. POPULATION and HOUSING CENSUS OF ETHIOPIA ADMINISTRATIVE REPORT Central Statistical Authority Addis Ababa. 2012.

[32] Central Statistical Agency (CSA). FEDERAL DEMOCRATIC REPUBLIC OF ETHIOPIA Demographic and Health Survey. 2016. 1–59 p.

[33] Hall SS (2006). Marital meaning. J Fam Issues.

[34] Eyo UE (2018). Divorce : causes and effects on children. Asian J Humanit Soc Stud.

[35] Scott ES (2000). Social norms and the legal regulation of marriage. Va Law Rev.

[36] Salvini S, Vignoli D (2011). Things change: Women’s and men’s marital disruption dynamics in Italy during a time of social transformations, 1970–2003. Demogr Res.

[37] Tian Y (1996). Divorce, gender role, and higher education expansion. High Educ.

[38] Applbaum KD (1995). Marriage with the proper stranger : arranged marriage in metropolitan Japan. Ethnology.

[39] Arficho AH (2020). Determinant analysis of divorce in Wolaita Sodo Town; in Case of Wadu Kebele, Snnpr. Ethiopia CCurr Tre Biosta Biometr.

[40] Tavakol M, Dennick R (2011). Making sense of Cronbach’s alpha. Int J Med Educ.

[41] Khidzir KAM, Ismail NZ, Abdullah AR. Validity and reliability of instrument to measure social media skills among small and medium entrepreneurs at Pengkalan Datu River. Int J Dev Sustain. 2018;7(3):1026–37. www.isdsnet.com/ijds

[42] Pannucci CJ, Wilkins EG (2010). Identifying and avoiding bias in research. Plast Reconstr Surg.

[43] Charan J, Biswas T (2013). How to calculate sample size for different study designs in medical research?. Indian J Psychol Med.

[44] Sreedharan J, Chandrasekharan S, Gopakumar A (2019). An Optimum sample size in cross sectional studies. Int J Sci Res Pap Math Stat Sci.

[45] Mishra P, Pandey CM, Singh U, Gupta A, Sahu C, Keshri A (2019). Descriptive statistics and normality tests for statistical data. Ann Card Anaesth.

[46] Di Leo G, Sardanelli F. Statistical significance: p value, 0.05 threshold, and applications to radiomics—reasons for a conservative approach. Eur Radiol Exp. 2020;4(1). 10.1186/s41747-020-0145-y PMC7064671 32157489

[47] Boateng EY, Abaye DA (2019). A review of the logistic regression model with emphasis on medical research. J Data Anal Inf Process.

[48] Oke JA, Akinkunmi WB, Etebefia SO (2019). Use of correlation, tolerance and variance inflation factor. Glob Sci J..

[49] Jain G (2019). Significance of marriage as social institution in Indian English writings. Soc Values Soc.

[50] Gujo FP (2019). Assessment on observance of essential conditions of marriage under customary marriage of sidama. Southern Ethiopia Beijing Law Rev.

[51] Mwambene L. Marriage under African customary law in the face of the Bill of Rights and international human rights standards in Malawi. African Hum Rights Law J. 2010;10(1):78–104. http://www.scielo.org.za/scielo.php?script=sci_arttext&pid=S1996-20962010000100005

[52] Theunis L, Schnor C, Willaert D, Van Bavel J (2018). His and her education and marital dissolution: adding a contextual dimension. Eur J Popul.

[53] Meeussen L, VanLaar C. Feeling pressure to be a perfect mother relates to parental burnout and career ambitions. Front Psychol. 2018;9(NOV). 10.3389/fpsyg.2018.02113 PMC6230657 30455656

[54] Hodgson N (2010). What does it mean to be an educated person?. J Philos Educ.

[55] Bell D (1997). Defining marriage and legitimacy. Curr Anthropol.

[56] Elfil M, Negida A (2019). Sampling methods in clinical research; an educational review. Arch Acad Emerg Med.

